# Draft genome sequence of the emerging fish pathogen *Flavobacterium tructae* strain S12

**DOI:** 10.1128/MRA.00488-23

**Published:** 2023-11-03

**Authors:** Shicheng Chen, Thomas P. Loch, Edward D. Walker

**Affiliations:** 1 Medical Laboratory Sciences Program, College of Health and Human Sciences, Northern Illinois University, DeKalb, Illinois, USA; 2 Department of Fisheries and Wildlife, College of Agriculture and Natural Resources, Michigan State University, East Lansing, Michigan, USA; 3 Department of Microbiology and Molecular Genetics, Michigan State University, East Lansing, Michigan, USA; University of Delaware College of Engineering, Newark, Delaware, USA

**Keywords:** *Flavobacterium tructae*, genome, fish, pathogenicity

## Abstract

The draft genome of *Flavobacterium tructae* strain S12, isolated from hatchery-reared Chinook salmon (*Oncorhynchus tshawytscha*) fingerlings, consisted of 5,695,942 bp, a G + C content of 35.6%, 4,775 predicted open reading frames, a putative type IX secretion system, collagenase, and hemolysin. *F. tructae* strains can be used as models for emerging *Flavobacterium* pathogens.

## ANNOUNCEMENT

*Flavobacterium tructae* strain T16^T^ was initially isolated from the kidneys of feral spawning adult Chinook salmon (*Oncorhynchus tshawytscha*) at Swan River Weir, Presque Isle County, MI, USA in October 2007, and strain S12 was isolated from the gills of cultured Chinook salmon fingerlings experiencing mortality at Thompson State Fish Hatchery, Schoolcraft County, MI, USA in May 2005 (1, 2). The bacterial isolates were acquired by plating infected tissues on Hsu-Shotts medium (3) and incubating aerobically at 22°C for up to 7 days. The obtained pure cultures were maintained in 20% glycerol at −80°C since isolation. The strains, when injected intraperitoneally, induced morbidity and mortality in Great Lakes Chinook salmon (4), suggesting that they were emerging facultative or opportunistic pathogens of these fish. Genome analysis of *F. tructae* strain T16^T^ showed that it carried a type IX secretion system, various virulence factors, and antimicrobial resistant genes (5). To further elucidate the molecular virulence mechanisms in these emerging flavobacterial pathogens, we report the genome sequence of *F. tructae* strain S12.

*F. tructae* strain S12 was grown in casitone-yeast extract [10 g of casitone per liter, 5 g of yeast extract per liter, and 8 mM MgSO_4_ in 10 mM Tris buffer (pH 7.6)] broth under aerobic conditions at 28°C. Genomic DNA extraction was performed using the Wizard Genomic DNA Purification Kit (Promega, Madison, WI, USA). Library construction was done using the Illumina TruSeq Nano DNA Library Preparation Kit (Illumina, San Diego, CA, USA). Paired-end next generation sequencing was carried out using Illumina MiSeq paired-end sequencing chemistry (2 × 250 bp) at the Research Technology Support Facility at Michigan State University. FastQC v0.11.9 was used to verify the sequencing quality (https://www.bioinformatics.babraham.ac.uk/projects/fastqc). The raw reads (2,149,940) were quality-trimmed to eliminate adapter sequences and low-quality ends, using CLC Genomics Workbench v.23.0 (https://digitalinsights.qiagen.com/) under default settings. Genome assembly using the same software produced 134 contigs ranging in size from 186 to 919,958 bp. CheckM v.1.0.18 (6) was used to check the quality of the assembled genome sequence with a completeness of 99.7% and contamination of 0.84%. After further cleaning of short sequences (<300 bp), the assembled genome had 87 contigs with an N50 scaffold size of 421,805 bp (more than 50-fold coverage). It comprised 5,695,942 bp nucleotides with a G + C content of 35.6%. The genome was automatically annotated by NCBI Prokaryotic Genome Automatic Annotation Pipeline (PGAAP, v.6.2) (7). No plasmids were detected using PlasmidFinder (8). There were at least 67 RNA sequences including 60 tRNAs, 4 rRNAs, and 3 noncoding RNAs in the genome.

*F. tructae* S12 genome carried genes encoding virulence factors such as a putative type IX secretion system (9), predicted collagenase, and putative hemolysin, which are conserved in most of the analyzed pathogenic *Flavobacterium* (9, 10). The putative conjugative transposon elements were identified by IslandViewer3 (11), and their sequences were very similar to CTnDOT by Blastn to the previously reported one (>90% identical) (5). This observation indicates that horizontal transfer of flavobacterial genes involved in antibiotic resistance and/or virulence factors is very likely in their hosts and native habitats (12, 13). Comparative genome analysis of *F. tructae* strains will be useful in future studies to determine virulence attributes as well as mechanisms that enhance its environmental adaptations and fitness.

## Data Availability

This Whole Genome Shotgun project has been deposited at DDBJ/ENA/GenBank under the accession JASTTY000000000. The version described in this paper is version JASTTY010000000. The raw data have been deposited in the SRA under the accession number SRR24847337.
